# Reproducibility of area at risk assessment in acute myocardial infarction by T1- and T2-mapping sequences in cardiac magnetic resonance imaging in comparison to Tc99m-Sestamibi SPECT

**DOI:** 10.1186/1532-429X-15-S1-P204

**Published:** 2013-01-30

**Authors:** Birgit Langhans, Eva Hendrich, Albert Schömig, Stefan Martinoff, Martin Hadamitzky

**Affiliations:** 1 Institute for Clinical Radiology, Ludwig-Maximilians-University Hospital Munich, Munich, Germany; 2 Klinik für Herz- und Kreislauferkrankungen, Deutsches Herzzentrum München, Munich, Germany; 3 Institut für Radiologie und Nuklearmedizin, Deutsches Herzzentrum München, Munich, Germany

## Background

Area at risk (AAR) is an important parameter for the assessment of the salvage area after revascularization in acute myocardial infarction (AMI). By combining AAR assessment by T2-weighted imaging and scar quantification by late gadolinium enhancement imaging cardiovascular magnetic resonance (CMR) offers a promising alternative to the "classical" modality of Tc99m-Sestamibi SPECT. Current T2 weighted sequences for edema imaging in CMR are limited by low contrast to noise ratios and motion artifacts. During the last years novel CMR imaging techniques for quantification of acute myocardial injury, particularly T1-mapping and T2-mapping, have attracted rising attention. But far no direct comparison between the different sequences in the setting of AMI and a validation against SPECT have been reported.

## Methods

We analyzed 14 patients undergoing primary coronary revascularization in AMI in whom both a pre-intervention Tc99m-Sestamibi-SPECT and CMR imaging at a median of 3.4 [3.3; 3.6] days after the acute event were performed. Size of AAR was measured by 4 different non-contrast CMR techniques on corresponding short axis slices: T2-weighted, fat-suppressed turbospin echo sequence (TSE), cine balanced FFE sequences in systolic phase (CINE), T2-mapping from T2-weighted balanced FFE sequences (T2-MAP) and T1-mapping (MOLLI). For each CMR sequence, the AAR was quantified by appropriate methods (absolute values for mapping sequences, comparison with remote myocardium or skeletal muscle for other sequences) and correlated with Tc99m-Sestamibi-SPECT. All measurements were performed on a 1.5 Tesla scanner.

## Results

The size of the AAR assessed by CMR was 28.7±20.9 % of left ventricular myocardial volume (%LV) for TSE, 15.8±10.3 %LV for CINE, 39.5±16.8 %LV for T2-MAP, and 33.0±14.9 %LV for MOLLI. AAR assessed by SPECT measured 41.6 ±20.7 %LV. Correlation analysis revealed best correlation with SPECT for T2-MAP at a R2-threshold of 62.5 milliseconds (ms; slope=0.86, Pearson's r=0.93), for MOLLI at R1-threshold of 1100 ms (slope 0.72, r=0.9), and for TSE at 2 SD above remote (slope=0.65, r=0.86). CINE at 2 SD above remote had a similar correlation (r=0.87) but significantly underestimated AAR (slope 0.35, p<0.001 for difference in AAR size).

## Conclusions

For the assessment of area at risk in AMI, the novel T2-mapping technique correlates best with SPECT size, T1-mapping with MOLLI and standard T2-weighted imaging show similar good correlations.

## Funding

none

**Figure 1:**
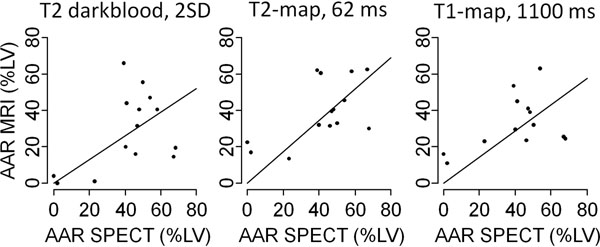
Correlation between CMR and SPECT studies for area at risk displayed as XY-plots in T2 darkblood (left), T2-map (middle), T1-map (right) sequences.

**Figure 2:**
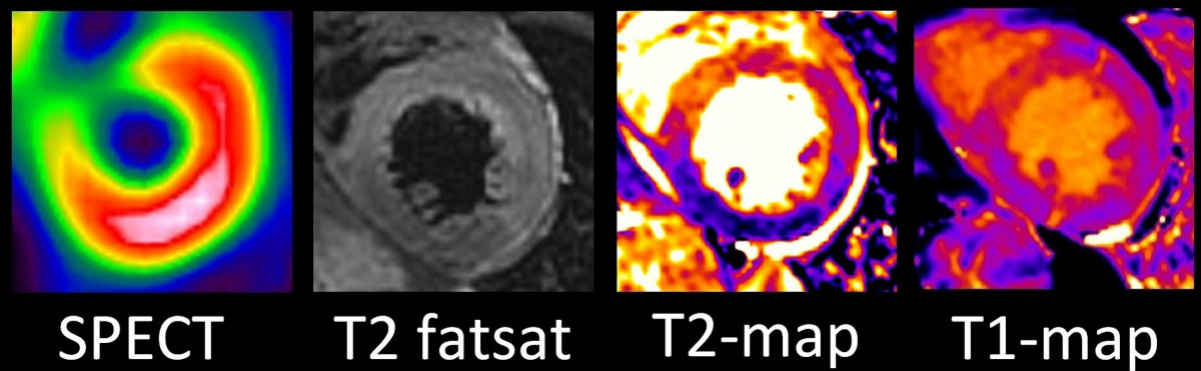
Area at risk in a corresponding short axis slices in SPECT, T2 fatsat, T2-map and T1-map images (from left to right).

